# MiR-150-5p contributes to unexplained recurrent spontaneous abortion by targeting *VEGFA* and downregulating the PI3K/AKT/mTOR signaling pathway

**DOI:** 10.1007/s10815-023-02959-w

**Published:** 2023-11-03

**Authors:** Wenyan Liao, Xin Deng, Guodong Chen, Juanli Yang, Yi Li, Li Li, Lili Zhong, Guangwei Tao, Jiafeng Hou, Mujun Li, Chengming Ding

**Affiliations:** 1 The First Affiliated Hospital, Department of Gynaecology and Obstetrics, Hengyang Medical School, University of South China, Hengyang, 421001 Hunan China; 2 Reproductive Medical Center, The First Affiliated Hospital of Guangxi Medical University, No. 6, Shuangyong Road, Nanning, 530021 Guangxi China; 3 The First Affiliated Hospital, Department of Hepatopancreatobiliary Surgery, Hengyang Medical School, University of South China, No. 69, Chuanshan Road, Hengyang, 421001 Hunan China

**Keywords:** Unexplained recurrent spontaneous abortions (URSA), miR-150-5p, VEGFA, PI3K/AKT/mTOR signaling pathway

## Abstract

**Purpose:**

The purpose of this study is to investigate the function of miR-150-5p in URSA.

**Method:**

Twenty-six chorionic villous tissues were collected to examine the expression of miR-150-5p and VEGFA by using quantitative polymerase chain reaction (qPCR) and western blot assay, respectively. Transwell assay was conducted to assess the migration and invasion ability of trophoblast cells. The dual-luciferase reporter assay was applied to determine the relationship between miR-150-5p and VEGFA in vitro. Relevant signaling pathway protein expression level was measured via western blot assay. Signaling transduction inhibitor LY294002 was used to block PI3K/AKT/mTOR signaling pathway. Finally, in vivo the effect of miR-150-5p on embryonic absorption rate was evaluated in mice.

**Results:**

Clinical samples revealed that miR-150-5p expression was significantly elevated in the villous tissues and serum of URSA patients. Moreover, the overexpressing of miR-150-5p could inhibit both HTR-8/SVneo cell and JAR cell migration, invasion, and restrained PI3K/AKT/mTOR signaling pathway by targeting VEGFA in vitro. This inhibitory effect of miR-150-5p could be reversed by overexpressing the gene of vascular epithelial growth factor A (*VEGFA*). In contrary, inhibition of miR-150-5p significantly enhanced migration, invasion ability of both HTR-8/SVneo and JAR cells, and also could stimulate PI3K/AKT/mTOR signaling pathway. This promoting effect of miR-150-5p could be ameliorated by LY294002 (PI3K inhibitor). Finally, after miR-150-5p overexpression in vivo, the embryo resorption rate in pregnant mice was increased significantly.

**Conclusions:**

Overall, these findings imply that miR-150-5p is among the key factors that regulate the pathogenesis of URSA.

**Supplementary Information:**

The online version contains supplementary material available at 10.1007/s10815-023-02959-w.

## Introduction

Recurrent spontaneous abortion (RSA) is the unintentional termination of two or more consecutive pregnancies before 20 weeks of gestation [1]. Globally, about 1–5% of all women suffer from RSA during reproductive age [2]. The cause of recurrent spontaneous abortions is very complex. Previous etiology studies mainly focused on chromosome factors, uterine anatomical structure abnormalities, infectious factors, endocrine abnormality, thrombophilia, abnormal immune function, environmental factors and other factors [3]. However, there is no identifiable cause in some cases, a condition called unexplained RSA (URSA) [4]. One possible cause may be related to deficient trophoblast cells. These embryo-derived cells often migrate and invade the uterine endometrium through a series of processes. Only when trophoblast cells successfully invade the endometrium, can a normal pregnancy be established. Hence, trophoblast cells are crucial to maintain pregnancy and any deficiency in their function could result to serious complications, such as pregnancy loss, preeclampsia, and intrauterine growth restriction [5].

miRNAs are a class of conserved noncoding small RNAs that function as posttranscriptional regulators of gene expression by binding to the 3′ untranslated region (UTR) of a target mRNA to either induce its degradation or inhibit its translation [6, 7]. It has been widely proven that miRNAs participate in a wide range of biological and pathological processes, such as cell differentiation, proliferation, apoptosis, angiogenesis, and even inflammation [8, 9]. And it was reported that miRNAs are closely related to various diseases, including spontaneous abortion (SA), of course. Therefore, from the perspective of genetics, miRNA is likely to participate in the occurrence of URSA by regulating gene expression. Qi Zhu found that miR-98 was highly expressed and could induce SA targeting GDF6 and FAPP2 to prevent proliferation, viability and migration of trophoblast cells in the villous tissues of SA, and Zhang CY also reported that MicroRNA-940 was up-regulated and promoted the occurrence of SA via targeting ZNF672 to inhibit the proliferation of trophoblast cells [10, 11]. Some studies have further reported the role of miRNA in RSA, for example: up-regulation of miR-365 may contribute to recurrent miscarriage by decreasing SGK1 expression [1]; MiR-184 is up-regulated in the villus and decidua of RSA patients [12]; high expression of miR-526b-5p could cause RSA via targeting c-Myc and Foxp1 to promote the proliferation, migration, and invasion of trophoblasts [13]. However, because there are few studies on miRNA in URSA, the potential mechanisms of miRNA regulating URSA progression remain unclear. Therefore, it is of great significance to explore the possible pathogenesis of URSA in the field of miRNA, which will provide a good theoretical basis for the diagnosis and treatment of URSA.

Notably, Zhao et. al. screened the miRNAs expression in RSA tissue and accidentally found that miR-150-5p was significantly highly expressed in RSA decidual tissues than normal decidual tissues [1]. However, further function and mechanism of miR-150-5p in RSA had not been previously elucidated. The ability of cytotrophoblast migration and invasion plays a key role in the process of embryo implantation. During the first trimester of pregnancy, trophoblast cells reach about 1/3 of the myometrium through its “physical invasion” ability. Then, the decidua tissue of the mother promotes the recasting and dilation of the spiral artery, so as to meet the nutritional and blood supply requirements of the mother and fetus during normal physiological pregnancy. This suggests that the physiological invasion function of trophoblast cells is a crucial prerequisite for successful implantation of fertilized egg and development of the embryo. Only when the trophoblasts invade the uterus decidua that a successful pregnancy can be established [14]. Inadequate migration and invasion of trophoblast cells can result in an impaired uterine spiral artery rebuilding and are implicated in RSA [15]. Given the increased expression of miR-150-5p in the decidual tissue of RSA, we want to check whether miR-150-5p could promote RSA by inhibiting trophoblast migration and invasion. To explore the influence of miR-150-5p on trophoblasts, we selected two kinds of cells: HTR-8/SVneo and choriocarcinoma JAR cells [16, 17], which are trophoblast model cells. In this study, we investigated the effects of miR-150-5p in vitro on cell migration, and invasive abilities. Furthermore, we demonstrated the detailed pathological mechanisms underlying URSA.

## Materials and methods

### Collection of tissue specimens

A total of 26 women aged 25–38 years with a history of two or more spontaneous abortions and 26 fertile women aged 24–37 years with one or more children and no history of spontaneous abortions were enrolled in this study between June 2019 and June 2020 in the Department of Obstetrics and Gynecology, the First Affiliated Hospital of University of South China. Baseline characteristics of all women were recorded (Table 1). Peripheral blood samples were taken from RSA patients and fertile women prior to undergoing induced abortion. Samples were centrifuged at 1500 rpm for 15 min after coagulation. The serum was then collected and stored at − 80°C until analysis. Villous tissues were obtained from cases of induced abortion between 6 and 10 gestational weeks. Cases of recurrent spontaneous abortion with known etiological causes including parental chromosomal, autoimmune, genital abnormalities such as uterus and cervix as well as presence of infection, diabetes mellitus, thyroid abnormalities and other endocrine abnormalities, smoking, drinking and other bad habits were excluded in this study. All patients provided informed consent regarding the use of their samples. One part of the samples was immediately snap-frozen in liquid nitrogen and preserved at − 80 °C until use. And the other part was immediately fixed in 4% neutral formalin for further immunohistochemical study. This study was approved by the Research Ethics Committee of the First Affiliated Hospital of University of South China (No. 20190511). This study was in agreement with the Declaration of Helsinki.

**Table 1:** Comparison of the general data of the two groups of pregnant women

	URSA (*n* = 26)	Normal (*n* = 26)	*P* value
Age (year)	33.96 ± 3.46	32.42 ± 3.00	0.094
Gestation age	8.38 ± 1.02	7.80 ± 1.23	0.072
Body mass index(BMI) (kg/m^2^)	20.78 ± 1.61	20.10 ± 1.03	0.075

### Cell culture and treatment

**Fig. 1:**
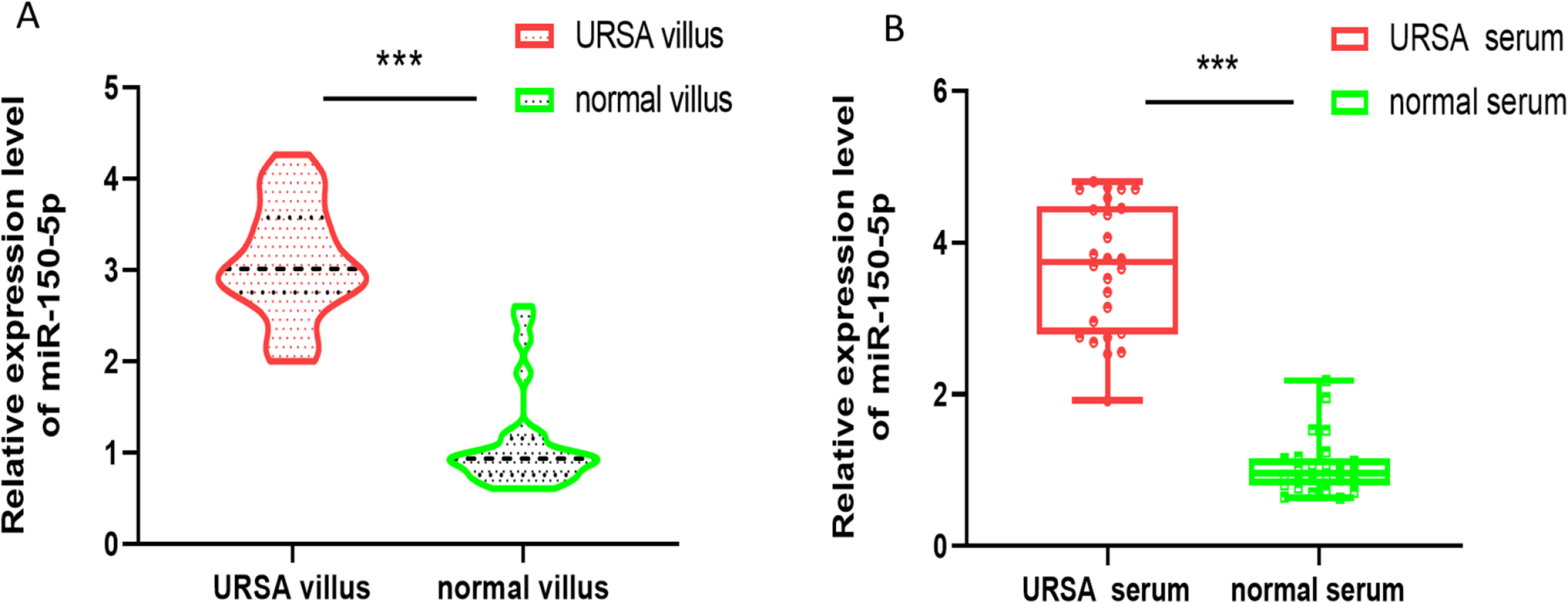
**High expression of miR-150-5p in the villous tissue of URSA.** The expression level of miR-150-5p in villous tissues of URSA and normal villous tissues were measured by qRT-PCR. ( *** *P*-value < 0.001)

Trophoblast HTR-8/SVneo and HEK-293 T cells were purchased from Shanghai Zhong Qiao Xin Zhou Biotechnology Co., Ltd. (Shanghai, China). JAR, JEG3 and BeWo cells were acquired as gift by Dr. Lingjie Deng in The First Affiliated Hospital of Guangxi Medical University (Nanning, China). HTR-8/Svneo cell line was shown to contain a heterogeneous population of both trophoblast and mesenchymal cells and was the most widely used cell line for EVT functions, so we selected HTR-8/Svneo as one of the trophoblast model cells. Additionally, in our pre-experiment, JAR, BeWo and JEG-3 were transfected at the same time, and it was found that JAR had higher transfection efficiency (Knock down efficiency: JEG3 45.57% vs JAR 40.37% vs BeWo 47.34%), so we chose JAR for the subsequent experiments (Please see the following Fig. 1S). HTR-8/SVneo cells and JAR cells were seeded in RPMI 1640 medium (Gibco, Carlsbad, CA, USA) supplemented with 10% fetal bovine serum (FBS, Gibco) whereas HEK-293 T cells were cultured in DMEM (Gibco) medium containing 10% FBS. All cells were incubated with the condition of 5% CO_2_ at 37 °C. miR-150-5p mimics/inhibitor and their negative control (NC mimics/NC inhibitor), small interference RNA targeting VEGFA (siVEGFA-1, siVEGFA-2) and their corresponding negative controls (si-NC) were purchased from Ribobio (Guangzhou, China). VEGFA full length cDNA was cloned into pcDNA3.1 vector. Due to its high efficiency and convenience, Lipofectamine 2000 (Invitrogen, Thermo Fisher Scientific, USA) was used for cell transfection according to the manufacturer’s instruction. LY294002 (Beyotime, China) was used as a PI3K inhibitor at the concentration of 20 μM. Cells were pre-treated with LY294002 for 2 h before indicated assays were performed. LY294002 was dissolved in Dimethylsulfoxide (DMSO), and we used DMSO as a Control.

### Cell migration and invasion analysis

HTR-8/SVneo cells and JAR cells transfected with miR-150-5p mimics, inhibitor, or their negative controls were placed on the top chamber of 24-well plate (Corning, New York, NY, USA) with or without matrigel (BD Biosciences, New York, NY, USA). After 24 h of incubation, the migrant or invasive cells that had attached to the lower surface of membrane were fixed with methanol for 30 min and then stained with 0.1% crystal violet for 30 min. Cells were counted using a microscope and then calculated the relative migration rate or invasion rate.

### Total RNA extraction and quantitative real-time PCR (qRT-PCR)

The total RNA was extracted according to the TRIzol Reagent manual (Invitrogen), followed by reverse transcription into cDNA with the miRcute miRNA First-Strand cDNA synthesis Kit (Tiangen, Beijing, China) following the manufacturer’s protocol. qRT-PCR was carried out using a Power SYBR Green PCR Master Mix (Applied Biosystems, USA). For *VEGFA* mRNA, cDNA was synthesized using PrimeScript™ reagent kit with gDNA Eraser (Takara, Japan), and qRT-PCR was analyzed using SYBR Premix Ex Taq kit II (Takara, Japan). The relative expression of miRNA or mRNA was analyzed using the 2^−ΔΔ^CT method. All results were normalized to GAPDH or U6 genes. The following primers were used: miR-150-5p (F: 5′-GCGTCTCC CAACCCTTGTA-3′ and R: 5′-AGTGCAGGGTCCGAGGTATT-3′), internal control U6 (F:5′-CTCGCTTCGGCAGCACA-3′ and R: 5′-AACGCTTCA CGAATTTGCGT-3′), *VEGFA* (F: 5′-TGGCTCACTGGCTTGCTCTA-3′and R: 5′-ATCCAACTGCACCGTCACAG-3′) and GAPDH (F: 5′-GGTGGTCTCC TCTGACTTCAA-3′ and R: 5′-GTTGCTGTAGCCAAATTCGTTGT-3′).

### ELISA assay

The VEGFA serum level was determined using human VEGFA ELISA kit (R&D Systems, Minneapolis, MN, USA) according the manufacturer’s instructions. The level of VEGFA was expressed in pg/ml, and optical density (OD) values were evaluated at 450 nm. According to OD value and standard curve calculation to determine the concentration.

### Dual-luciferase reporter assays

The *VEGFA* 3′-UTR that possessed a miR-150-5p binding site was inserted into the luciferase-reporter vector, pmirGLO (Promega, Madison, WI, USA), downstream of the firefly luciferase gene. HEK-293 T cells were co-transfected with pmirGLO-*VEGFA*-3′UTR-WT and pmirGLO-*VEGFA*-3′UTR-Mut reporter plasmids and miR-150-5p mimics/inhibitor and NC mimics/NC inhibitor. Forty-eight hours posttransfection, dual-luciferase reporter assay (Promega, Madison, WI, USA) was performed to measure the relative luciferase activity. Luciferase activity was normalized to Renilla luciferase activity.

### Immunohistochemistry

The villous tissues were fixed with formaldehyde and then embedded in paraffin and cut into sections of 4–5 μm. VEGFA antibody was diluted to 1:1200. The sections were added with primary antibody and kept at 4℃ overnight, and the secondary antibody at room temperature for 1 h. The sections were visualized under an inverted microscope at 400 × magnification. Images were analyzed using Image J software (National Institutes of Health, USA). The expression intensity of VEGFA in villous tissues was analyzed by integrated optical density (IOD).

### Western blot assay

Proteins were extracted by RIPA lysis buffer mixed with phenylmethyl sulfonylfluoride (PMSF) and phosphatase inhibitors. After adding SDS-PAGE loading buffer, samples were incubated at 100 °C for 5 min to denature the proteins. Proteins were separated in sodium dodecyl sulphate–polyacrylamide gel (SDS-PAGE) and transferred onto a poly-vinylidene fluoride (PVDF) membrane. The membrane was then blocked with 5% non-fat milk for 1 h. Primary antibodies were incubated overnight at 4°C and then incubated with horseradish peroxidase-conjugated secondary antibody (Beyotime, Shanghai, China). The primary antibodies were VEGFA, AKT, p-AKT, PI3K, p-PI3K, mTOR, p-mTOR antibody (1/1000 dilution, Cell Signaling Technology, USA), and GAPDH antibody (1/1000 dilution, Beyotime Biotechnology, China). The membrane was stained using the enhanced chemiluminescence system (Minichemi610, China). The quantification of protein was done by digital analyses of protein bands using the ImageJ software (National Institutes of Health, USA).

### Animal experiments

BALB/c (8–10 weeks) were obtained from Hunan SJA Laboratory Animal Co., Ltd (ChangSha, Hunan, China), and kept in a specific pathogen-free animal facility. The procedures involved in the mouse studies were approved by the Research Ethics Committee of the First Affiliated Hospital of University of South China.miR-150 agomir or negative control agomir (miR agomir NC) were purchased from Ribobio Biotech. Female mice were injected with miR-150 agomir or miR agomir NC through a tail vein every 3 days until death. After the first injection, female mice were mated with male mice in cages. Eleven days after vaginal plug was observed, female mice were sacrificed and the embryonic absorption rate was calculated.

### Statistical analysis

All experiments were performed more than three times. Statistical analyses were carried out using GraphPad Prism 8 (GraphPad Software, Inc., La Jolla, CA, USA) and SPSS version 19.0 software (IBM, Armonk, NY, USA). Student’s *t*-test or Mann–Whitney *U* test was used to estimate the differences between two groups. One-way analysis of variance (ANOVA) (LSD test) was used to estimate the differences between Multiple groups. Spearman's rank correlation coefficient was performed to do correlation analysis. A *p* value of < 0.05 was regarded as statistically significant.

## Results

### MiR-150-5p is highly expressed in the villous tissues and serum of URSA patients

We detected miR-150-5p expression in human villous specimens of URSA by qRT-PCR. The result showed that the expression of miR-150-5p in villous and serum from URSA is significantly higher than healthy controls (Fig. 1A, B).

### MiR-150-5p inhibits the migration, and invasion of trophoblast cell

**Fig. 2:**
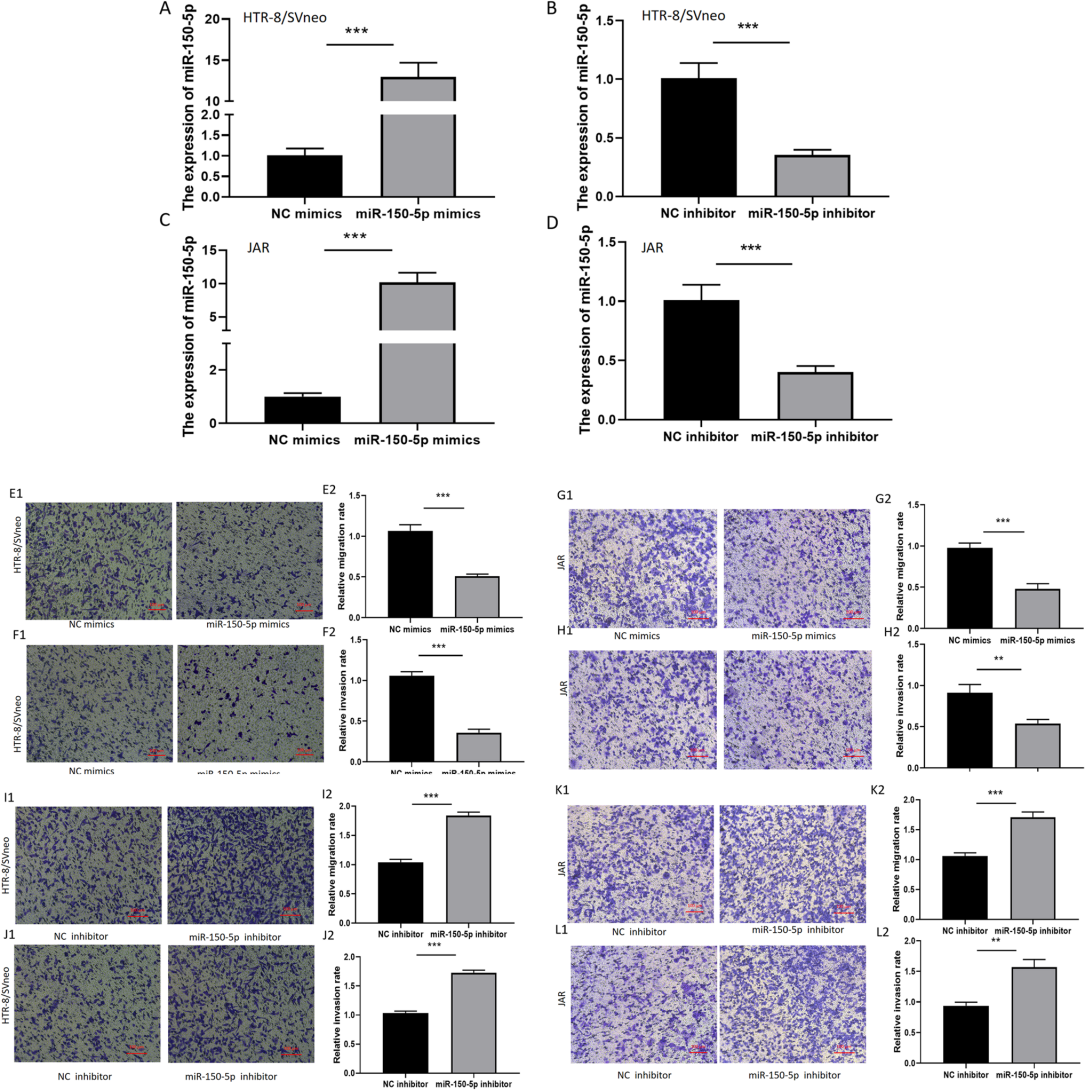
**MiR-150-5p inhibits the migration, and invasion of trophoblast cells.** (**A**–**D**) The transfection efficiency was measured by qRT-PCR. (**E**–**L**) Transwell assay was conducted to measure the cell migration and invasion ability. (** *P*-value < 0.01, ***P-value < 0.001)

To explore the biological functions of miR-150-5p on trophoblast cells, we transfected HTR-8/SVneo and JAR cells with miR-150-5p mimics/miR-150-5p inhibitor and their corresponding negative control. The transfection efficiency was measured by qRT-PCR (Fig. 2A, B and C, D), indicating that miR-150-5p was indeed overexpressed and knockdown in trophoblast cells. The overexpression of miR-150-5p can suppress migration and invasion (Fig. 2E, F and G, H) of trophoblast cells, whereas its knockdown can promote the migration and invasion (Fig. 2I, J and K, L) of trophoblast cells.

### *VEGFA* is a direct target gene of miR-150-5p

**Fig. 3:**
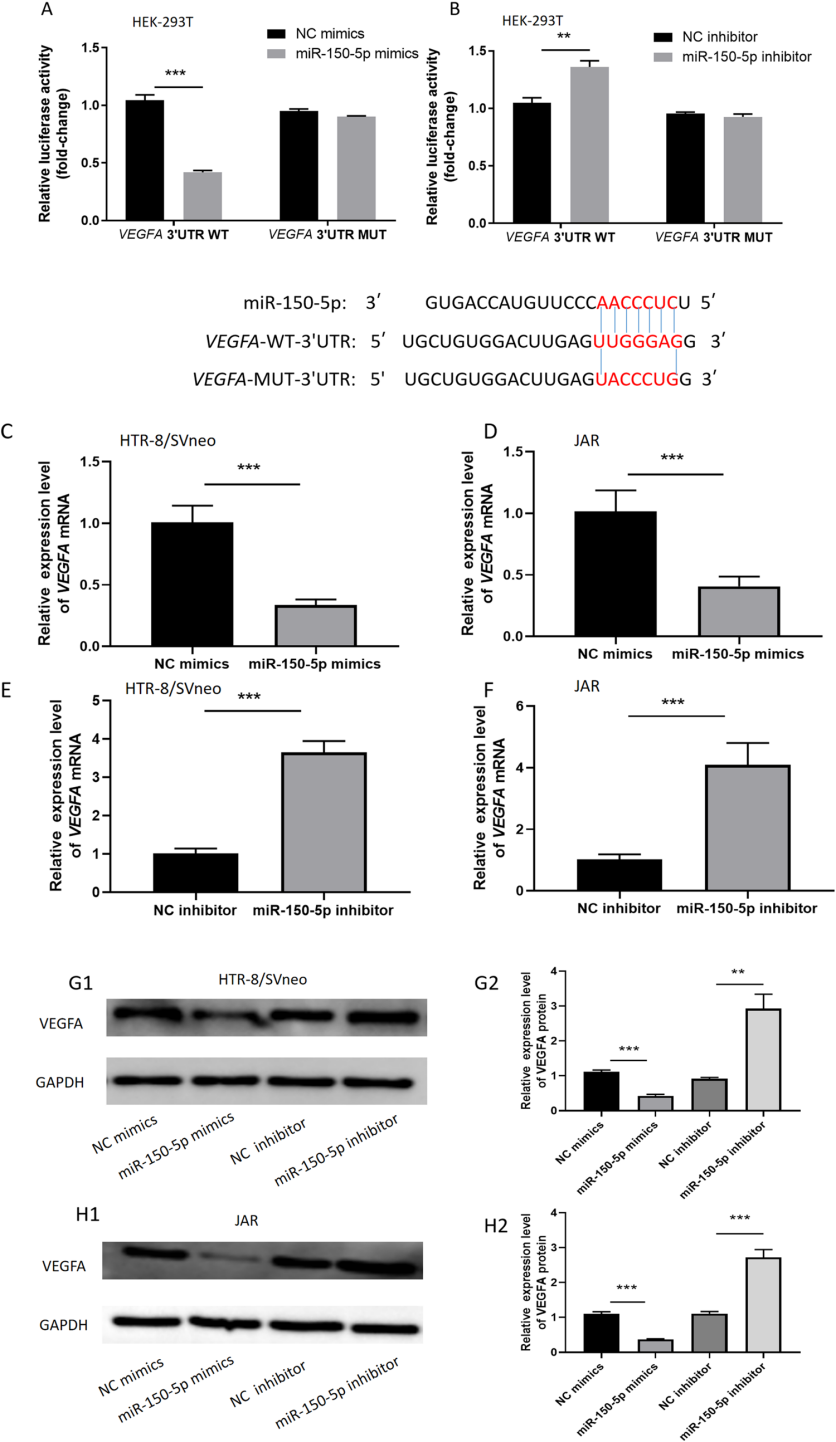
***VEGFA* is a direct target gene of miR-150-5p.** (**A**–**B**) In HEK-293 T cells, NC mimics/NC inhibitor or miR-150-5p mimics/miR-150-5p inhibitor was cotransfected with pmirGLO-*VEGFA*-3′UTR-WT or pmirGLO-*VEGFA*-3′UTR-Mut, and luciferase reporter assay was performed 48 h later. (**C**–**F**) After transfect with miR-150-5p mimics/miR-150-5p inhibitor in trophoblast cells, the expression of *VEGFA* mRNA was analyzed by qRT-PCR analyses. (**G**–**H**) After transfect with miR-150-5p mimics/miR-150-5p inhibitor in trophoblast cells, the expression of VEGFA protein was analyzed by Western blot. (**P-value < 0.01, ***P-value < 0.001)

The miRDB (http://mirdb.org/) and Tarbase (http://carolina.imis.athena-innovation.gr/diana_tools/web/index.php?r=tarbasev8%2Findex/) databases were used to search for potential target genes of miR-150-5p. From the overlapping target genes retrieved from these two databases, we selected *VEGFA* for experimental validation. To determine whether *VEGFA* is a direct target gene of miR-150-5p, we performed dual-luciferase reporter assays. The overexpression of miR-150-5p in HEK-293 T cells obviously reduced the luciferase activity of pmirGLO-*VEGFA*-3’UTR-WT but with no significant effect on the luciferase activity of pmirGLO-*VEGFA*-3′UTR-Mut (Fig. 3A). Upon knockdown of miR-150-5p, we found that the luciferase activity of pmirGLO-*VEGFA*-3′UTR-WT was increased but the luciferase activity of pmirGLO-*VEGFA*-3′UTR-Mut has no significant difference (Fig. 3B). Additionally, the *VEGFA* mRNA in HTR8/SVneao cells and JAR cells dramatically decreased following the overexpression of miR-150-5p (Fig. 3C and D). In contrast, the opposite phenomenon was observed upon inhibiting miR-150-5p; that is, the knockdown of miR-150-5p resulted to increased *VEGFA* mRNA levels (Fig. 3E and F). Last but not least, when overexpression or knockdown miR-150-5p, the change of VEGFA protein level was consistent with *VEGFA* mRNA (Fig. 3G and H).

### Low expression of *VEGFA* in the villous tissues and serum of URSA patients

**Fig. 4:**
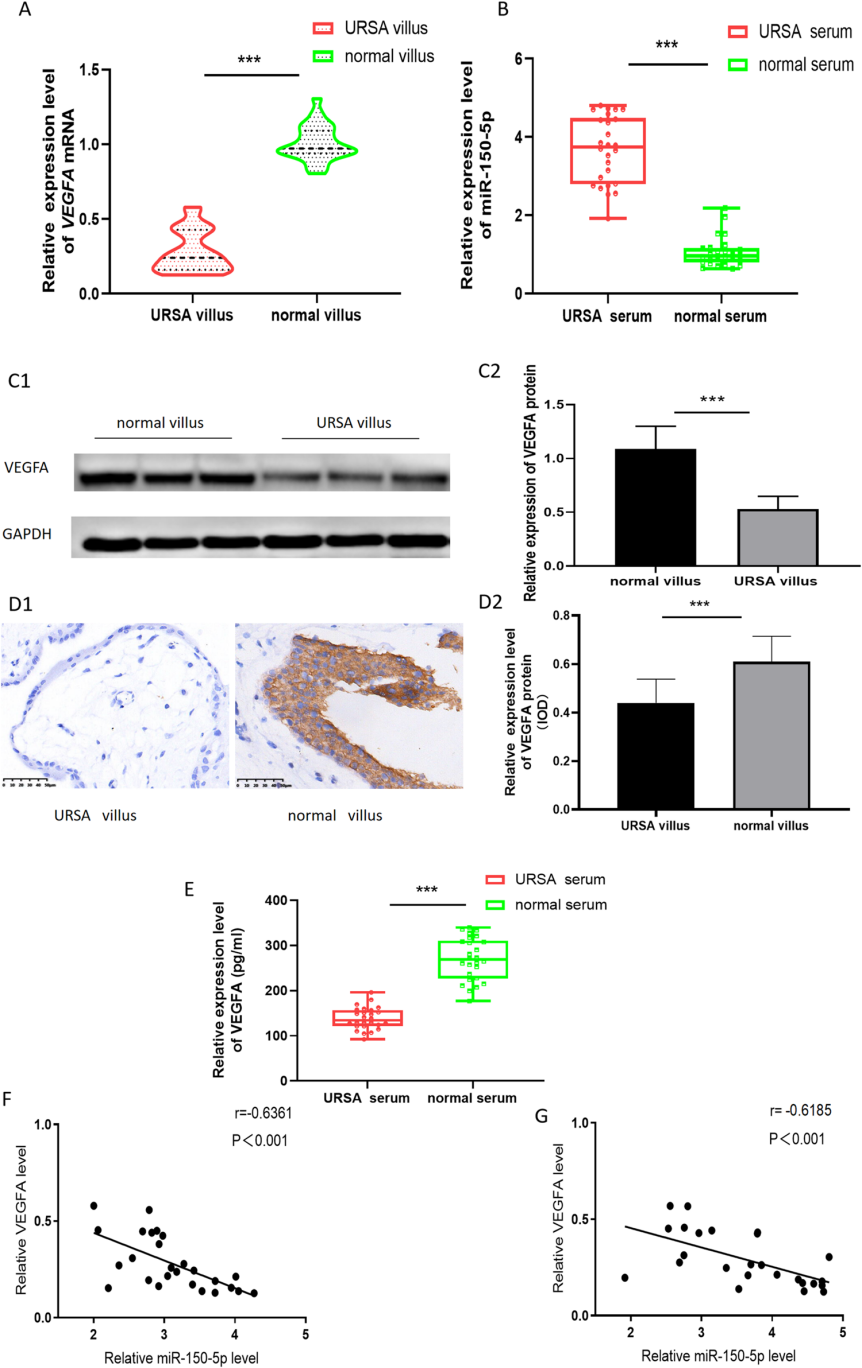
**Low expression of VEGFA in the villous tissues of URSA patients.** (**A**) The expression of *VEGFA* mRNA was analyzed by qRT-PCR analyses. (**B**) The expression of VEGFA protein was analyzed by Western blot. (**C**) The expression of VEGFA protein was analyzed by Immunohistochemistry. (**D**) The correlation between miR-150-5p and *VEGFA* mRNA was analyzed by Spearman’s rank correlation coefficient (***P-value < 0.001)

To further characterize the functional significance of miR-150-5p in URSA via modulating VEGFA expression, we determined the differential expression of VEGFA of tissue samples from URSA patients and healthy controls. We found that the expressions of *VEGFA* mRNA and protein levels were significantly down-regulated in the villous tissues and serum of URSA patients (Fig. 4A, B and C, D, E). Meanwhile, miR-150-5p expression in the villous tissues is negatively correlated with *VEGFA* mRNA level (Fig. 4F, G).

### *VEGFA* promotes trophoblast cell migration and invasion

**Fig. 5:**
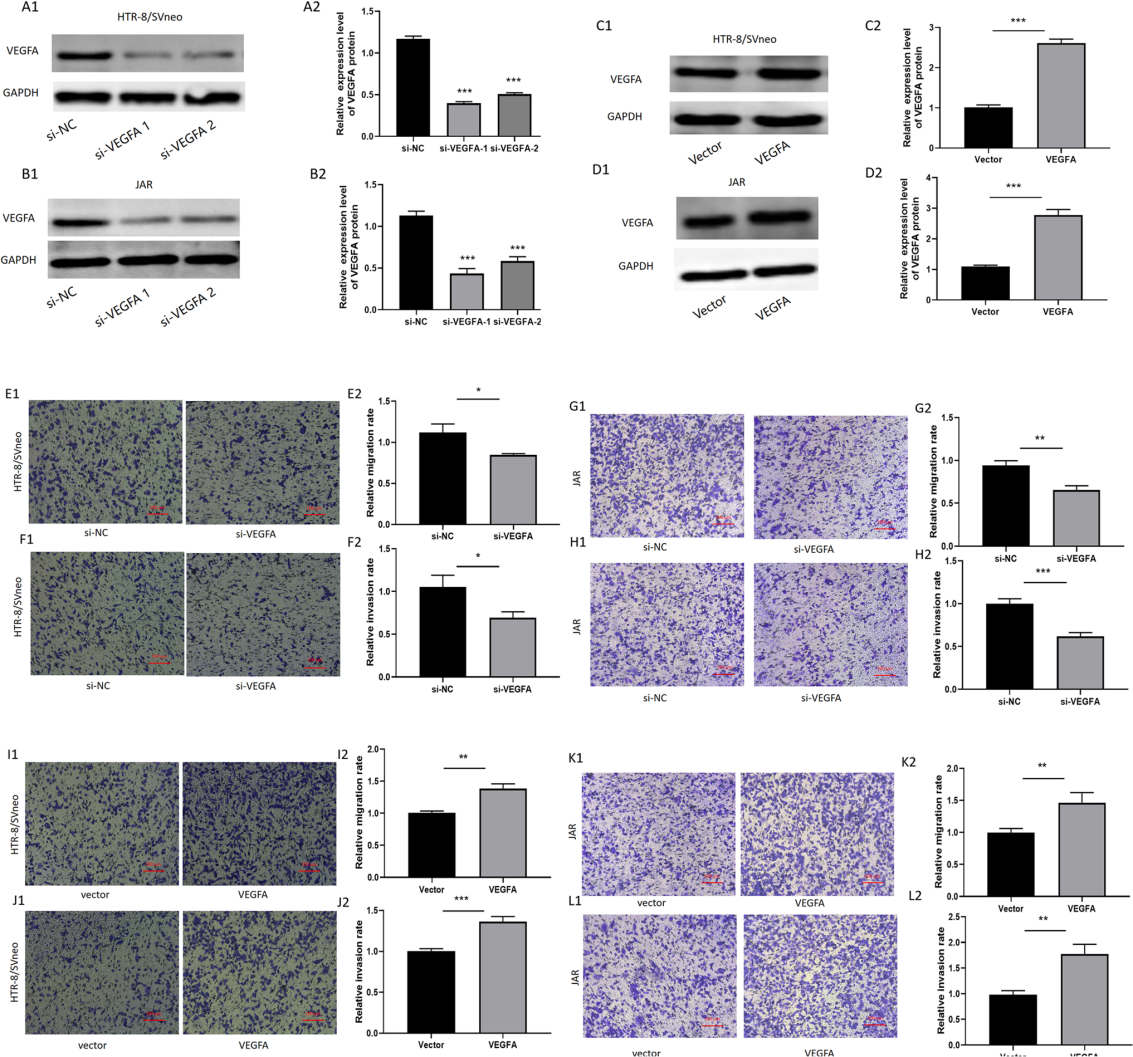
**VEGFA promotes trophoblast cell migration and invasion.** (**A**–**B**) After VEGFA siRNAs (siVEGFA-1, siVEGFA-2) were transfected individually, relative expression of VEGFA in trophoblast cells was detected by Western blot assays. The results showed that siVEGFA-1 is more effective, so we selected siVEGFA-1 for all subsequent experiments. (**C**–**D**) Overexpression of VEGFA was validated by Western blot. (**E**–**L**) Transwell assay was conducted to measure the cell migration and invasion ability. (*P-value < 0.05, **P-value < 0.01,***P-value < 0.001)

Next, we also knocked down or overexpressed VEGFA expression using siRNA or overexpression plasmid in HTR-8/SVneo and JAR cells (Fig. 5A, B and C, D). We found that the knockdown of VEGFA could markedly suppress trophoblast cell migration and invasion (Fig. 5E, F and G, H). Meanwhile, the overexpression of VEGFA can promote the migration and invasion ability of trophoblast cell (Fig. 5 I, J and K, L). These data imply that miR-150-5p may exert its biological function by inhibiting VEGFA expression.

### Overexpression of *VEGFA* could reverse the inhibitory effect caused by miR-150-5p

**Fig. 6:**
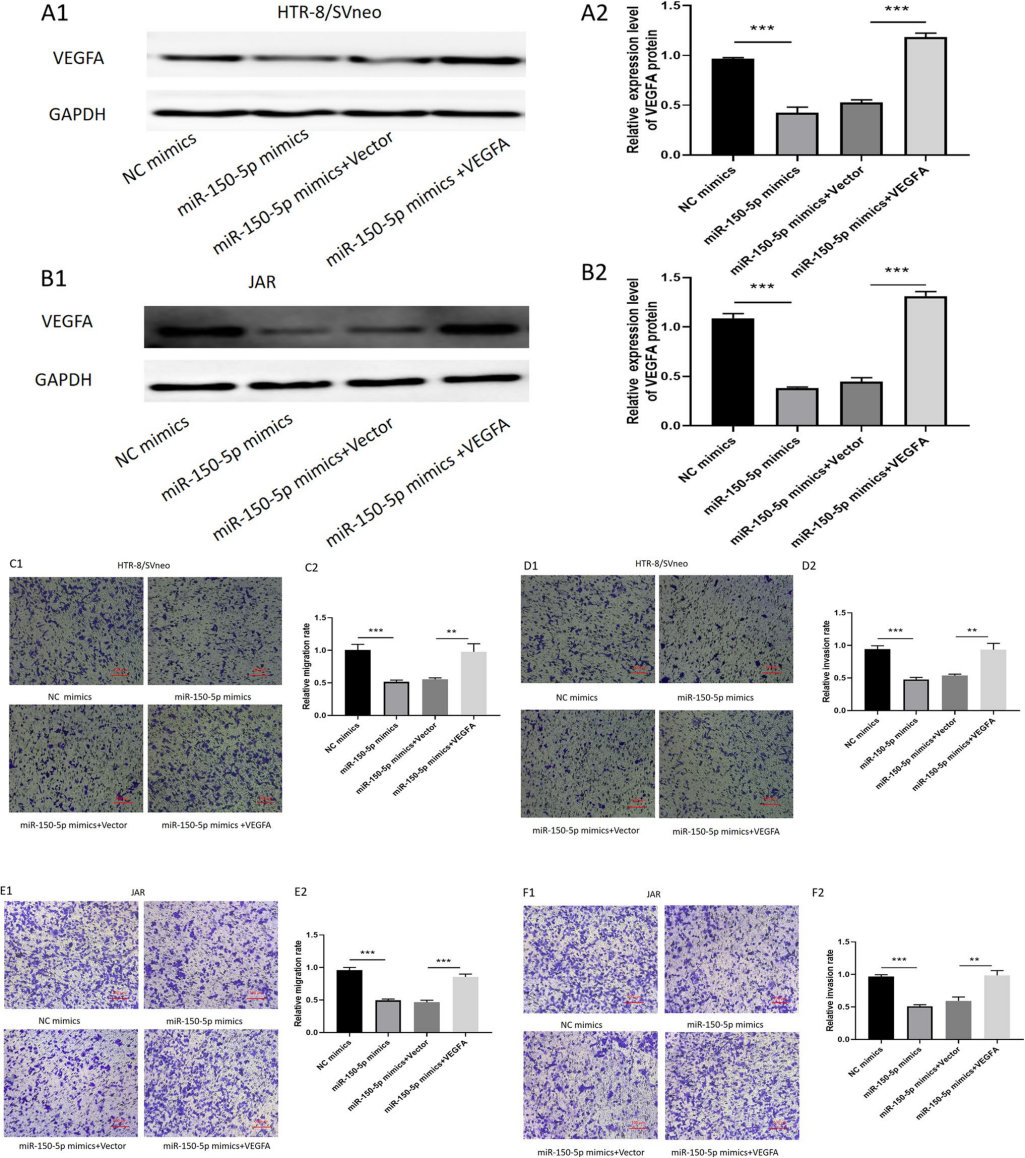
**Overexpression of VEGFA could reverse the inhibitory effect caused by miR-150-5p.** (**A**–**B**) VEGFA protein was verified by Western blotting in transfected HTR-8/SVneo and JAR cells. (**C**–**F**) Cell migration and invasion were detected by transwell migration and invasion assay ( **P-value < 0.01,***P-value < 0.001)

To investigate whether miR-150-5p suppresses migration and invasion through modulating the expression of VEGFA, we cotransfected miR-150-5p mimics and pcDNA 3.1-VEGFA. The VEGFA expression was verified by Western blotting (Fig. 6A, B). Through transwell migration and invasion assays, we found that VEGFA could partially reverse the suppression of migration and invasion (Fig. 6C, D and E, F) caused by miR-150-5p overexpression. These data indicated that VEGFA was not only a direct target gene but also a functional mediator of miR-150-5p in URSA.

### MiR-150-5p negatively modulates PI3K/AKT/mTOR signaling pathway by inhibiting *VEGFA*

**Fig. 7:**
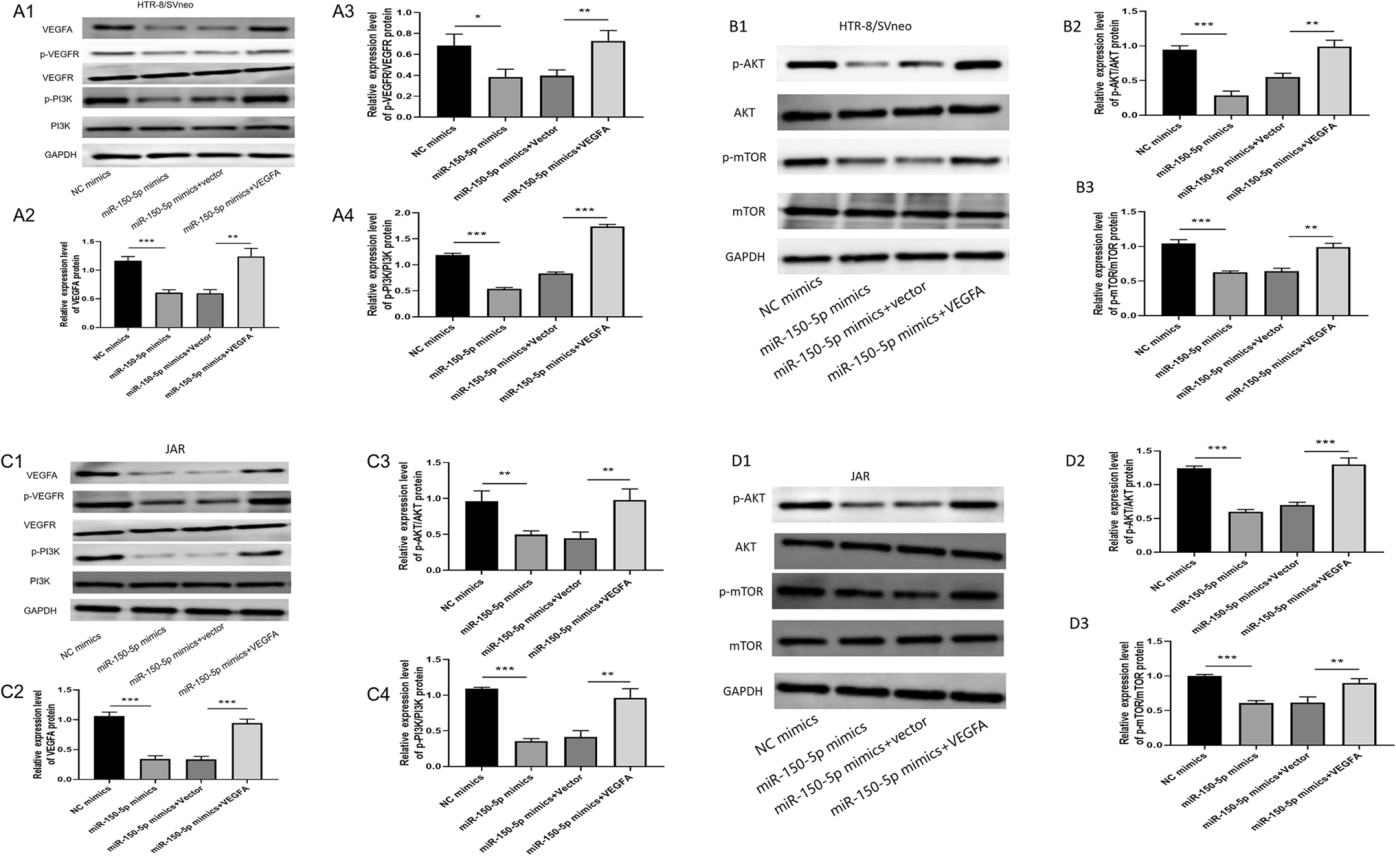
**MiR-150-5p negatively modulated PI3K/AKT/mTOR signaling pathway by directly inhibiting VEGFA.** (**A**–**D**) Western blot was used to measure the expression of VEGFA,PI3K, p-PI3K, AKT, p-AKT, mTOR, p-mTOR in transfected trophoblast cells. GAPDH was used as a loading control ( **P-value < 0.01,***P-value < 0.001)

Accumulating evidences have demonstrated that VEGFA can activate PI3K and its downstream targets AKT and mTOR [18, 19]. This activation of PI3K/AKT/mTOR pathway plays crucial roles in cancer progression and survival, particularly during cell invasion and migration [19–21]. Since our previous results had linked miR-150-5p with VEGFA, we further performed further experiments to confirm whether miR-150-5p promotes URSA through VEGFA-mediated inactivation of PI3K/AKT/mTOR signaling pathway. We found that the expression level of VEGFA can be decreased via overexpression of miR-150-5p in HTR-8/SVneo and JAR cells. This resulted to significant suppression of p-PI3K, p-AKT, and p-mTOR. Next, we found that compared to the miR-150-5p mimics group, the cotransfection of miR-150-5p mimics and VEGFA overexpression plasmid in trophoblast cells could at least partially rescue the expression of p-PI3K, p-AKT and p-mTOR (Fig. 7A, B and C, D).

### LY294002 as PI3K inhibitor reversed the effect of miR-150-5p inhibitors

**Fig. 8:**
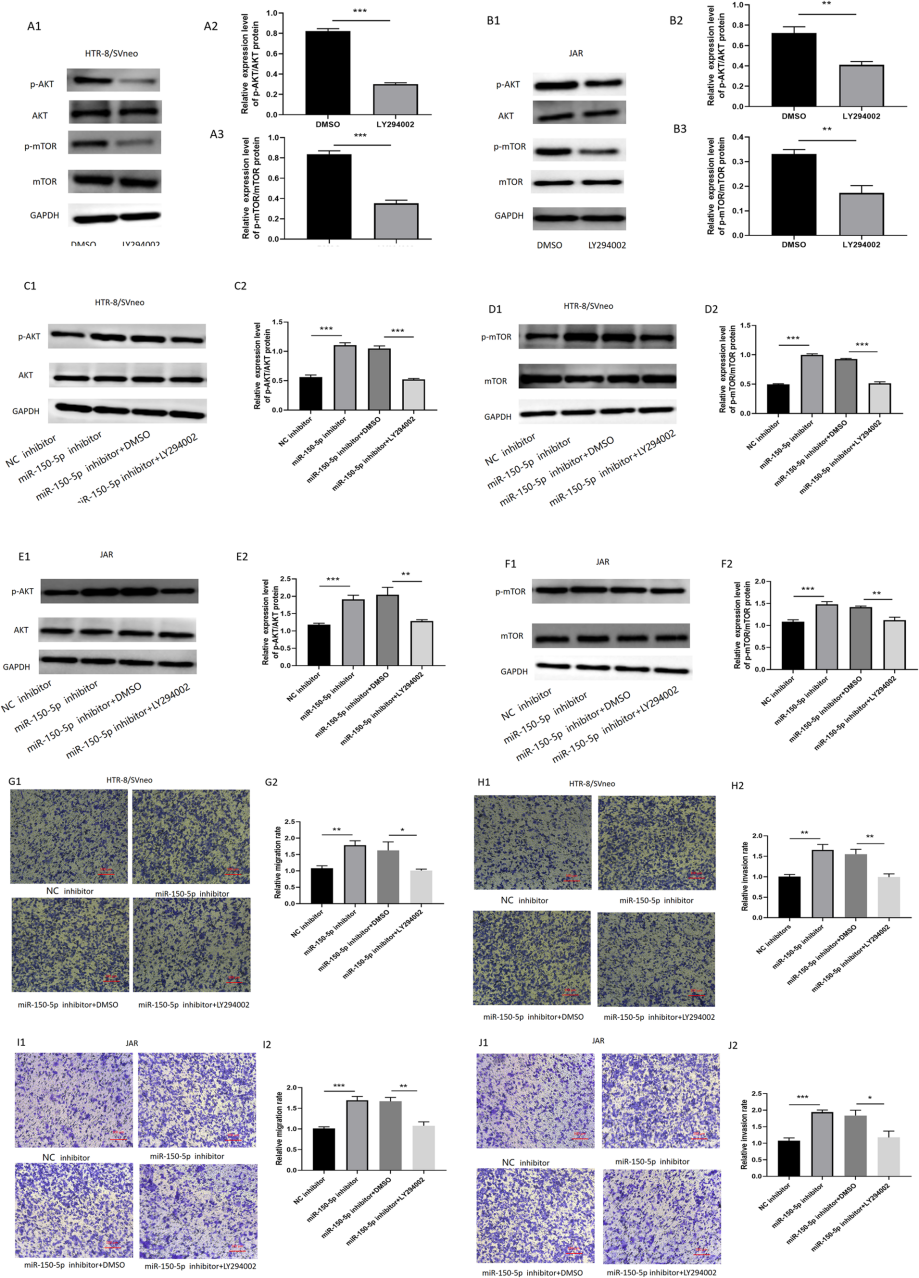
**LY294002 as PI3K inhibitor reversed the effect of miR-150-5p inhibitors.** (**A**–**F**) The protein levels of AKT, p-AKT and mTOR, p-mTOR were examined by Western blot in trophoblast cells with indicated treatments. (**G**–**J**) Transwell assay was conducted to measure the cell migration and invasion ability. (*P-value < 0.05, **P-value < 0.01, ***P-value < 0.001)

To further illustrate the regulatory function of miR-150-5p in PI3K/AKT /mTOR signaling pathway, we treated HTR-8/SVneo and JAR cells with LY294002, a PI3K inhibitor. LY294002 can significantly inhibit the expression of p-AKT and p-mTOR (Fig. 8A, B). Interestingly, the expression levels of p-AKT and p-mTOR in cells treated with miR-150-5p inhibitor were significantly increased, but such were reversed after treatment with LY294002. (Fig. 8C, D and E, F). As illustrated in Fig. 8G, H and I, J, the migration and invasion capacity showed that miR-150-5p inhibitor could dramatically increase cell migration and invasion, and after administration of LY294002 at the same time, the cell migration and invasion were significantly reduced. Altogether, these data demonstrated that miR-150-5p acts as a negative modulator of PI3K/AKT /mTOR signaling pathway via targeting VEGFA.

### MiR-150-5p increases the embryo resorption rate in vivo

**Fig. 9:**
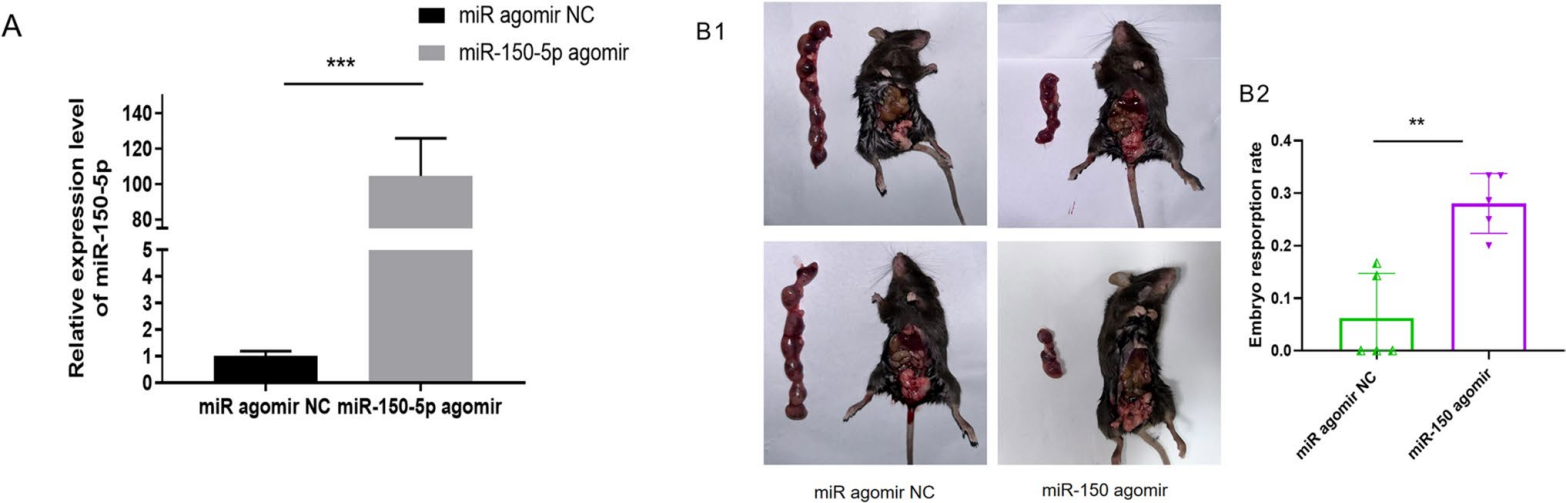
MiR-150-5p increases the embryo resorption rate in vivo. **A** In two groups of mice, we administered negative control（miR agomir NC) and miR-150-5p agomir respectively, and qRT-PCR was conducted to detect the expression of miR-150-5p in the peripheral blood of these mice. **B** Gross morphology of pregnant uterus and quantification of embryo resorption rate in each group (**P-value <0.01, ***P-value <0.001)

To verify the effect of miR-150-5p in vivo, injection of miR-150-5p agomir or negative control (miR agomir NC) into the female BALB/c mice, and qRT-PCR was performed to detect the expression of miR-150-5p in the peripheral blood. The result suggested that the expression of miR-150-5p was indeed increased in mice injected with miR-150-5p agomir (Fig. 9A). Thereafter, as shown in Fig. 9B, embryo resorption rate of mice injected with miR-150-5p agomir was significantly higher than that of the control group.

## Discussion

URSA is a common and complex disease with unclear mechanism among couples of reproductive age. Currently, the generally accepted etiology of URSA is inadequate migration and invasion of trophoblast cells. In our study, we found that miR-150-5p could promote URSA via preventing the migration and invasion of trophoblast cells, and then preliminarily explored the related mechanism. MiRNAs are important post-transcriptional regulators of complementary mRNA targets and have been implicated in the pathophysiology of URSA [22]. While it is possible that certain proteins are altered affecting specific cell signaling pathways, the other inadequately described is the role of microRNAs (miRNAs) in RSA. Liu et al. reported that miR-93 inhibits trophoblast cell proliferation and promotes cell apoptosis by targeting BCL2L2 in RSA [23]. Zhu et al. demonstrated that miR-16 inhibits fetomaternal angiogenesis and causes recurrent spontaneous abortion by targeting vascular endothelial growth factor (VEGF) [24]. Yet, the specific regulatory mechanism governing their invasion function is still not fully understood.

In the past decade, more and more miRNAs have been identified to play significant regulatory functions in various biological processes [25, 26]. miRNAs can exert their function by modulating their target genes via elevating translational repression or mRNA degradation [27]. For instance, miR-150-5p is aberrantly expressed in various malignancies, including non-small cell lung cancer (NSCLC), colorectal cancer, and Juvenile myelomonocytic leukemia (JMML) [28–30]. In several solid tumors, miR-150-5p has been recognized as a suppressor. In particular, miR-150-5p exerts its inhibitory function in colorectal cancer [31]. In melanoma, miR-150-5p negatively regulates the oncoprotein RAB9A [32]. Similarly, miR-150 downregulates MYB to suppress proliferation, migration, and invasion of melanoma cell [33]. With these evidences, miR-150-5p is therefore involved in the proliferation, migration, invasion and apoptosis of different tumor cells, and plays a regulatory role in different tissues. However, whether miR-150-5p functions in unexplained RSA (URSA) the same way as described in other disease states, and the potential mechanisms of miR-150-5p regulation of URSA progression, is still unclear.

Here, we explored the function of miR-150-5p in URSA. Specifically, we provided evidences that ectopic expression of miR-150-5p significantly suppressed the migration and invasion of trophoblast cells. Moreover, we confirmed that *VEGFA* is a direct target gene of miR-150-5p. This is consistent with the conclusion that Chen et al. found that VEGFA is the target gene of miR-150-5p in colorectal cancer [34]. It is reported that during the initial stages of pregnancy, VEGF participates in placental angiogenesis during embryogenesis [35]. Dysregulated VEGF expression may therefore elicit the onset of placental pathologies including preeclampsia, early pregnancy loss and intrauterine growth restriction [36]. Thus, the altered VEGF expression can be associated to early pregnancy loss and likely contributes to the etiology of RSA [37]. In our study, the expression of VEGFA in the villous tissues of URSA patients had obvious negative correlation with miR-150-5p. Our functional experiments showed that knockdown of VEGFA significantly inhibits the migration and invasion of trophoblast cells, while overexpression of VEGFA promotes the migration and invasion. In addition, overexpression of VEGFA could reverse the inhibitory effect caused by miR-150-5p. These results provide evidence that miR-150-5p functions as an upstream regulator of VEGFA expression, affecting cell migration and invasion of trophoblasts.

PI3K/AKT/mTOR signaling pathway is one of the main downstream effectors of VEGFA, participating in different cellular biological processes [38, 39]. A previous study has reported that mTOR signaling pathway may be related to trophoblast cell invasion and migration. Lower expression of PVT1 can promote RSA via inhibiting mTOR pathway-mediated autophagy [40]; ATL1 inhibits the proliferation and invasion of trophoblast cells via inhibition of the mTOR signaling pathway [41]; MGST1 alleviates the oxidative stress of trophoblast cells induced by hypoxia/reoxygenation and promotes cell proliferation, migration, and invasion by activating the PI3K/AKT/mTOR pathway [42]. We further examined whether miR-150-5p promotes URSA initiation by inactivating the PI3K/AKT/mTOR signaling pathway. Our data demonstrate that miR-150-5p overexpression could significantly decrease p-PI3K, p-AKT, and p-mTOR. Co-overexpression of miR-150-5p and VEGFA weakened the inhibitory effect. On the other hand, the knockdown of miR-150-5p can increase the activity of p-PI3K, p-AKT, and p-mTOR, but this effect was diminished in the influence of PI3K inhibitor (LY294002).

Taken together, our data suggest that miR-150-5p is highly expressed in URSA, which inhibits the migration and invasion of trophoblast cells via VEGFA-mediated inactivation of PI3K/AKT/mTOR signaling pathway (Figure S1). Because our work is still a preliminary report of miRNAs in URSA, the exact regulatory mechanism and the related signaling pathways of miR-150-5p require further studies. However, we provided here a new insight into the mechanism of URSA.

## Conclusions

We investigated the changes in miR-150-5p expression and their association with URSA, putting forward a new molecular mechanism to elaborate the miR-150-5p targeting VEGFA downregulates PI3K/AKT/mTOR signaling pathway to inhibit the migration and invasion of trophoblast cells, mediates the genesis of URSA.

### Supplementary Information

Below is the link to the electronic supplementary material.

Fig. S1 Proposed signaling pathways underpinning the essential role of miR-150-5P in URSA. miR-150-5p is highly expressed in URSA, which significantly decreased the level of VEGFA, then downregulated PI3K/ AKT/mTOR signaling pathway, thereby inhibiting the migration and invasion of trophoblast cells. (PNG 325 kb)

High Resolution (TIF 3707 kb)

Fig. 1S The Knock down efficiency of miR-150-5p in three cell lines (PNG 92 kb)

High Resolution (TIF 177 kb)

## Data Availability

The datasets used and/or analyzed during the current study are available from the corresponding author on reasonable request.
