# Supplementary figures and images for: MiR-150-5p contributes to unexplained recurrent spontaneous abortion by targeting VEGFA and downregulating the PI3K/AKT/mTOR signaling pathway
Source: J Assist Reprod Genet. 2023 Nov 3;41(1):63–77. doi: 10.1007/s10815-023-02959-w (PMC10789717; doi:10.1007/s10815-023-02959-w)

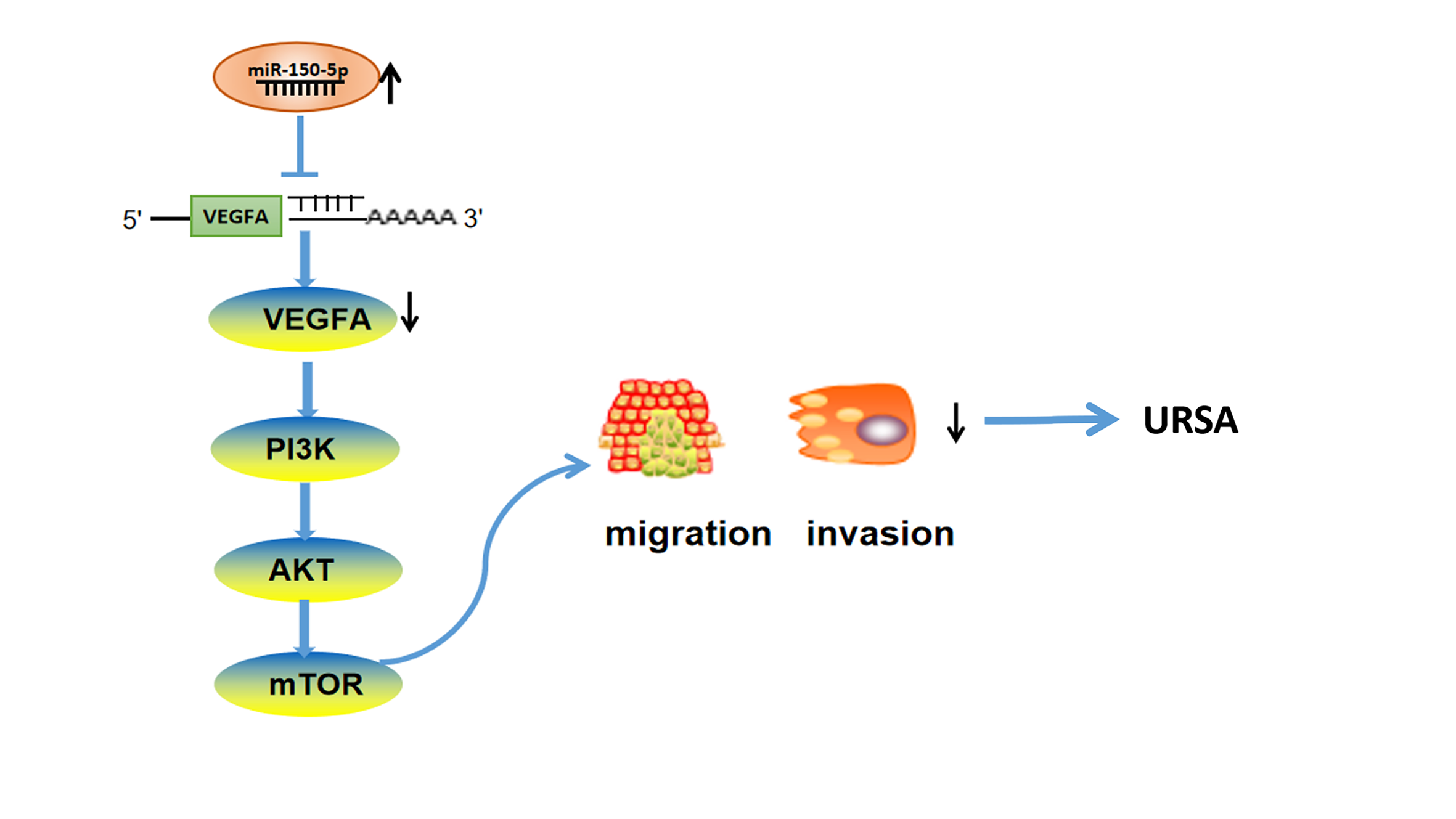

Supplement: Supplementary file 1 — Proposed signaling pathways underpinning the essential role of miR-150-5P in URSA. miR-150-5p is highly expressed in URSA, which significantly decreased the level of VEGFA, then downregulated PI3K/ AKT/mTOR signaling pathway, thereby inhibiting the migration and invasion of trophoblast cells. (PNG 325 kb) [file 10815_2023_2959_Fig10_ESM.png]

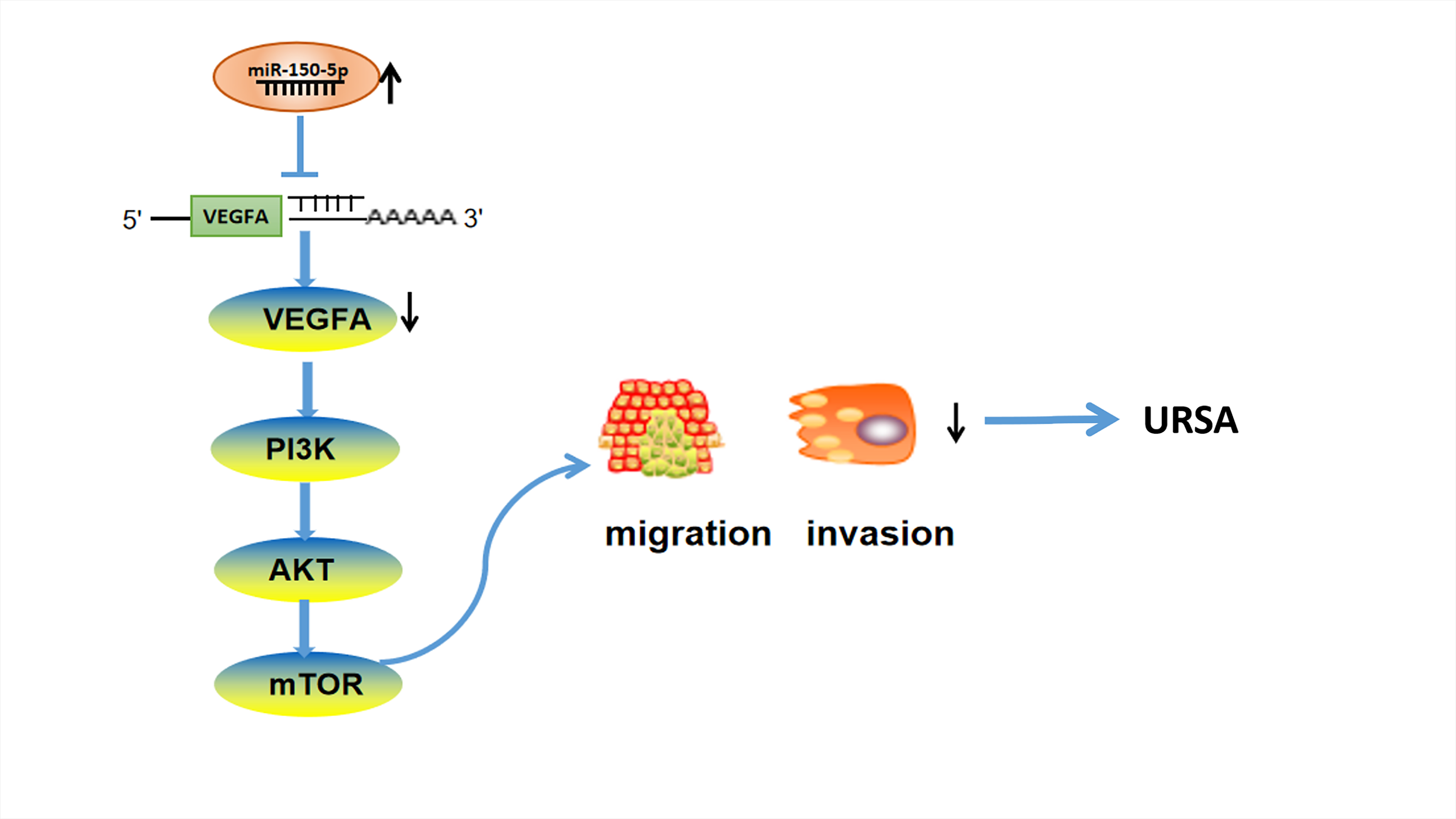

Supplement: Supplementary file 2 — High Resolution (TIF 3707 kb) [file 10815_2023_2959_MOESM1_ESM.tif]

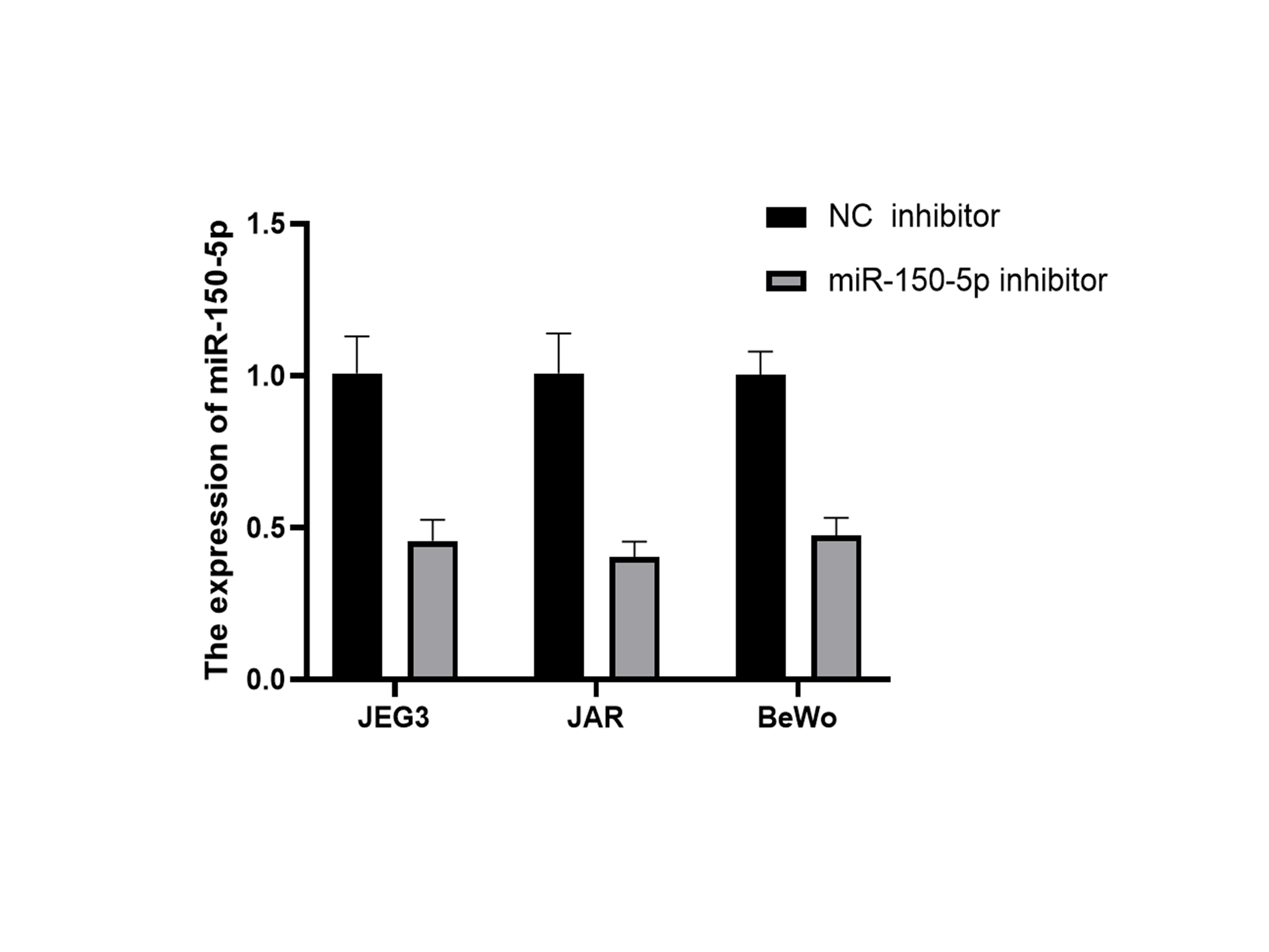

Supplement: Supplementary file 3 — The Knock down efficiency of miR-150-5p in three cell lines (PNG 92 kb) [file 10815_2023_2959_Fig11_ESM.png]

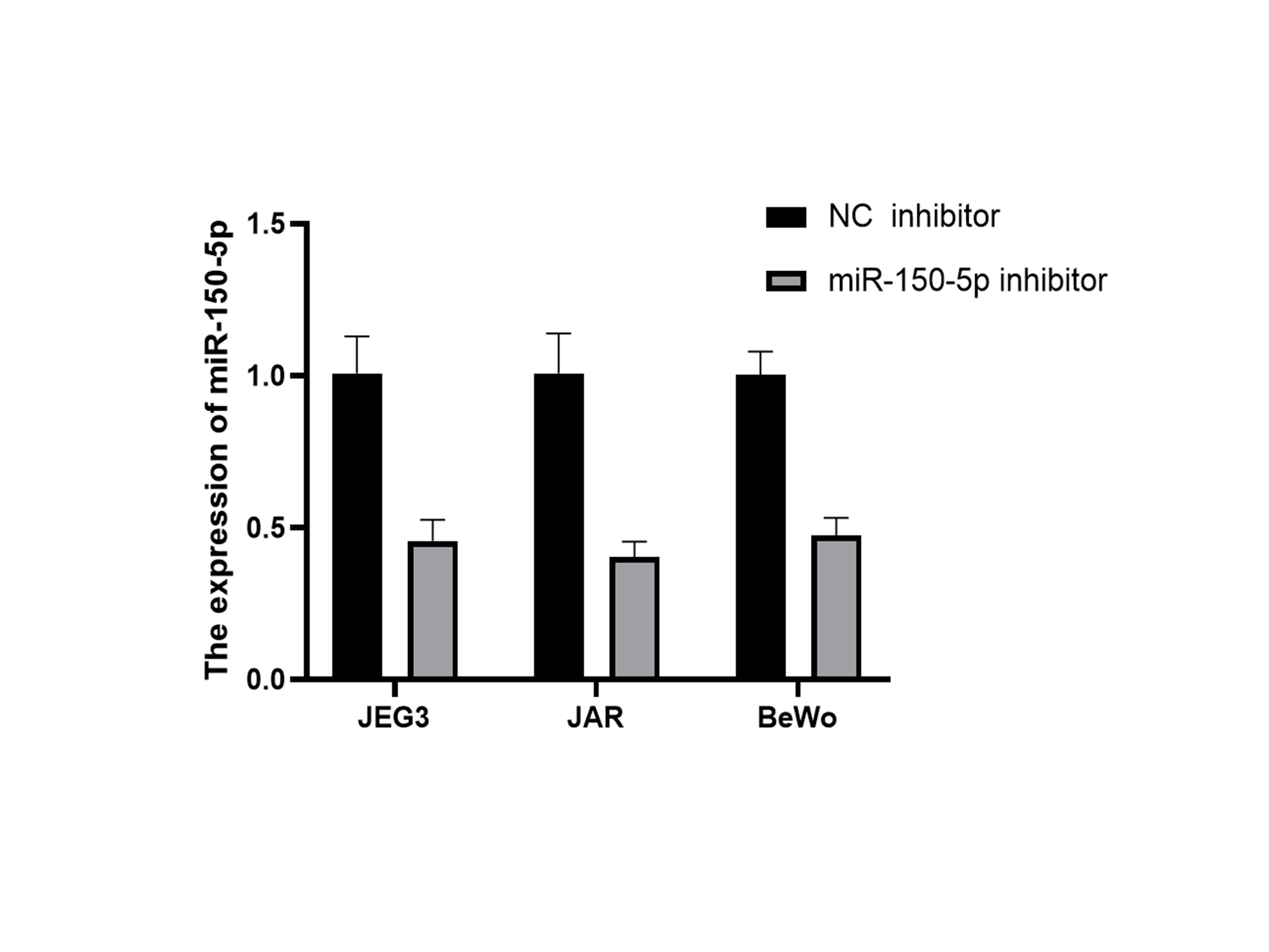

Supplement: Supplementary file 4 — High Resolution (TIF 177 kb) [file 10815_2023_2959_MOESM2_ESM.tif]
